# Commonsense knowledge in cognitive robotics: a systematic literature review

**DOI:** 10.3389/frobt.2024.1328934

**Published:** 2024-02-29

**Authors:** Jan-Philipp Töberg, Axel-Cyrille Ngonga Ngomo, Michael Beetz, Philipp Cimiano

**Affiliations:** ^1^ Center for Cognitive Interaction Technology, Bielefeld University, Bielefeld, Germany; ^2^ Joint Research Center on Cooperative and Cognition-enabled AI, Bielefeld, Germany; ^3^ DICE Group, Paderborn University, Paderborn, Germany; ^4^ Institute for Artificial Intelligence, University of Bremen, Bremen, Germany

**Keywords:** commonsense knowledge, cognitive robotics, systematic literature review, knowledge representation, semantic reasoning

## Abstract

One of the big challenges in robotics is the generalization necessary for performing unknown tasks in unknown environments on unknown objects. For us humans, this challenge is simplified by the commonsense knowledge we can access. For cognitive robotics, representing and acquiring commonsense knowledge is a relevant problem, so we perform a systematic literature review to investigate the current state of commonsense knowledge exploitation in cognitive robotics. For this review, we combine a keyword search on six search engines with a snowballing search on six related reviews, resulting in 2,048 distinct publications. After applying pre-defined inclusion and exclusion criteria, we analyse the remaining 52 publications. Our focus lies on the use cases and domains for which commonsense knowledge is employed, the commonsense aspects that are considered, the datasets/resources used as sources for commonsense knowledge and the methods for evaluating these approaches. Additionally, we discovered a divide in terminology between research from the knowledge representation and reasoning and the cognitive robotics community. This divide is investigated by looking at the extensive review performed by Zech et al. (The International Journal of Robotics Research, 2019, 38, 518–562), with whom we have no overlapping publications despite the similar goals.

## 1 Introduction

Robots have the potential to support us in a number of activities. Recently, there has been a massive adoption of cost-efficient robots that support us in house cleaning (e.g., vacuuming) and gardening (e.g., lawn mowing) activities. Moreover, research in household robotics has led to robots being able to clean breakfast tables (Kazhoyan et al., 2021), or prepare drinks (Sung and Jeon, 2020) and pizzas (Joublin et al., 2024). Yet, the ability of robots to support us in complex everyday tasks is still very limited. In particular, they break down in open world situations where they are challenged by new and underdetermined tasks, new environments or new objects about which they lack knowledge (Bronfman et al., 2021; Ding et al., 2023).

This gap between underdetermined tasks and the robot body motion that accomplishes the task has to be bridged through the robot’s knowledge and its reasoning capabilities. This challenge is the core of the research field of *cognitive robotics*, where knowledge representation and reasoning techniques are employed to support “autonomous robot [s] in a dynamic and incompletely known world” (Levesque and Lakemeyer, 2008, p. 869). A substantial part of these techniques and capabilities used to increase the robustness of cognitive robots in everyday tasks concerns the robot’s *commonsense knowledge (CSK)*. This knowledge has the benefit of “enhancing the quality of the plans […] as well as avoiding human involvement when making decisions” (Pradeepani et al., 2022, p. 159) and allows them “to ask and retrieve the right answers from available knowledge” (Salinas Pinacho et al., 2018, p. 132).

As the name suggests, CSK in humans is understood as “information that people usually take for granted and, hence, normally leave unstated” (Cambria et al., 2012, p. 3582), which increases the difficulty for automatic acquisition and deployment. Regarding the cognitive robotics domain, we follow the definition provided by Gupta and Kochenderfer (2004), which focuses on knowledge about human desires, physics, and causality, as well as knowledge about objects with their locations, properties and relationships. In general, knowledge about human desires correlates to the concept of *Intuitive Psychology* from Lake et al. (2017), with which an agent understands that other agents have a mental state similar to their own which they can express and interpret to understand their intentions and goals. Both knowledge about physics and knowledge about causality are covered by the concept of *Intuitive Physics*, also from Lake et al. (2017). This type of knowledge is focused on primitive physical concepts like the calculation of physically possible trajectories or the tracking of objects over time. With causality, also the knowledge about physical connections between objects and actions is covered. So, for example, CSK focused on causality would help a robot to understand the (physical) consequences of moving an object.

As a general example, consider a cognitive robot tasked with the preparation of a bowl of cereals for breakfast, a task that a human could perform without explicit planning. However, many of the implicitly known aspects for the human are challenges for the robot, since it needs to know that “a bowl of cereal” implies the use of milk or what constitutes a container to be used as the bowl or where to find the cereal in its environment. Without CSK that provides answers to these challenges, the robot would, e.g., search the whole kitchen for milk instead of starting with the most probable location (the fridge) or it would not understand that a found container could be used as the bowl.

By equipping cognitive robots with CSK, their robustness when interacting in open worlds is increased. However, the application of the concept of CSK to the cognitive robotics domain has received relatively limited research attention. There are no surveys or comparable studies performed to analyze the coverage of CSK for cognitive robotics. Since cognitive robotics are increasingly breaching into human domains, we perform a systematic literature review providing researchers and practitioners alike with an overview for CSK in cognitive robotics. For this literature review, we follow the principles and guidelines provided by Kitchenham and Charters (2007), Okoli (2015) and Page et al. (2021). To increase repeatability and traceability of our review, we track our progress in a review protocol and collect all intermediate results. All of these additional resources are available in our GitHub repository^1^.

To guide our research, we formulate the following four research questions, focusing on different aspects of CSK. Our motivation for these questions stems from the need to comprehensively understand the landscape of CSK utilization in cognitive robotics research. By addressing these research questions, we aim to uncover insights into the various use cases, specific aspects considered (or overlooked) in CSK application, the prevalent datasets or resources in the field, and the diverse methods employed for assessing these approaches. This comprehensive examination is crucial in shaping our understanding of the current state and potential future directions of CSK integration in cognitive robotics.

**RQ1** For which use cases has the use of CSK been considered in cognitive robotics research?
**RQ2** Which aspects of CSK have been considered? Which aspects of CSK have received less consideration?
**RQ3** Which datasets or resources are mainly considered in cognitive robotics as a source for CSK?
**RQ4** What methods are employed to assess the approaches? Which CSK datasets or resources are utilized in these evaluations?

To summarize our results, concerning **RQ1** we find that most use cases occur in the household domain and focus on objects and their relations to the environment. This is corroborated by our results pertaining to **RQ2**, which we address by looking at what sorts of questions CSK is called upon to answer. We found that the most common CSK questions seek to connect an object to a specific location in its environment. Other important questions focus on object similarity, object affordances and tool substitution. Here, affordances describe possible ways for an agent to interact with the environment Bornstein and Gibson (1980). In general, questions focusing on objects are much more dominant than questions about interacting with humans or about physics or causality of actions. Concerning **RQ3**, we find that while specific sources such as ConceptNet (Speer et al., 2017) (Open-)Cyc (Lenat, 1995) or OMICS (Gupta and Kochenderfer, 2004) are used multiple times, there is no one single source that is employed in all or most CSK use cases. Regarding the evaluation method and data covered by **RQ4**, we found that most approaches either evaluate using a *Case Study* or an *Experiment*, predominantly in a simulated environment. Unfortunately, most of the evaluation data is not available online.

During our search for suitable publications, we were surprised to notice a lack of publications that focus on well established keywords like *affordance learning*. After manually analyzing this gap using another, similar review by Zech et al. (2019)–with which we have no overlapping publications–we hypothesize that the reason is a divide in terminology between research in the cognitive robotics community and in the knowledge representation and reasoning community. We further explore this divide and propose possible bridges to close this gap.

## 2 Related work

Commonsense and intuitive physics reasoning problems were driving forces for knowledge representation and reasoning in early stages of AI research (McCarthy, 1959; 1977; Schank and Abelson, 1975; Minsky, 1981; Hayes, 1990). This line of research was presented in textbooks (Davis, 1990; 2008a; Mueller, 2014) and further developed within its own research community (Davis, 2008a; b; Levesque et al., 2012; Davis and Marcus, 2015). In current AI research, CSK is used for question-answering (Talmor et al., 2019; Nguyen et al., 2021), knowledge base creation (Tandon et al., 2017), text interpretation (Bisk et al., 2020; Puri et al., 2023) and visual recognition (Zellers et al., 2019), to name a few.

While a large fraction of research problems were motivated through intuitive physics and physical agency, they were not sufficiently leveraged in cognitive robotics research. Another characteristic of CSK and reasoning is its hybrid nature. Commonsense reasoning includes a large number of specialized methods for prospection (Szpunar et al., 2014), part-based reasoning (Tenorth and Beetz, 2017), mental simulation (Hesslow, 2012), imagistic reasoning (Nanay, 2021), planning (Ghallab et al., 2016), and safe human-robot collaboration (Conti et al., 2022), which were investigated individually without being linked to the more general concept of commonsense. In addition, representations of actions as they are investigated in natural language processing, such as FrameNet (Baker et al., 1998), are of key importance for robotic commonsense (Vernon, 2022). Furthermore, robot cognitive architectures contribute to robot commonsense by focusing on cognitive capabilities (Vernon, 2014; Vernon, 2022).

Regarding previous reviews on the topic of commonsense knowledge in cognitive robotics, as far as we know, no direct previous publications exists. However, works by Paulius and Sun (2019) and Sun and Zhang (2019) survey general knowledge representation techniques employed for different domains and scenarios. The work by Paulius and Sun (2019) focuses on knowledge representation and its connection to learning techniques applied in service robots, covering general high-level as well as specialized representations. Similarly, Sun and Zhang (2019) reviews three types of knowledge representations for task planning in robotics: semantic networks, rules and logical knowledge representation.

The survey conducted by Thosar et al. (2018) focuses on different knowledge bases that are employed by service robots manipulating household objects. These knowledge bases are compared regarding their knowledge acquisition and representation as well as the mechanisms used for inference and symbol grounding. Another review by Buchgeher et al. (2021) that focuses on a specific type of knowledge representation, looks into the usage of knowledge graphs for industrial manufacturing and production systems. The authors analyse application scenarios, graph characteristics and meta information about the surveyed research publications.

Reviews by Olivares-Alarcos et al. (2019) and Manzoor et al. (2021) focus on ontology-based approaches for knowledge representation. The review conducted by Olivares-Alarcos et al. (2019) surveys the cognitive capabilities supported by different ontologies, and compares them using a proposed classification schema based on the underlying ontology language and hierarchy as well as the application domain of the ontology. The review by Manzoor et al. (2021), on the other hand, focuses specifically on the household, hospital, and industry domains, looking for concrete scenarios where the ontologies have been applied on real robots.

Lastly, the literature review by Zech et al. (2019) focuses on the concept of *actions* in the cognitive robotics domain by looking at their representation and providing a possible taxonomy for their classification. Based on the classification of 152 publications, the authors summarize open research challenges for increasing the maturity and usability of action representations. This review exemplifies the divide mentioned in Section 1 regarding the terminology used by researchers with a (cognitive) robotics background and researchers in the knowledge representation and reasoning domain, since some concepts covered by their taxonomy are semantically equivalent to concepts from the knowledge representation and reasoning domain without being explicitly connected.

The reviews and surveys presented here differ in the knowledge representation approach covered, the application domain, and whether the review is structured in a systematic way. The topic of commonsense knowledge itself is not covered by any of these reviews. Due to the importance of commonsense knowledge for cognitive robotics, we investigate its application domain, data sources, evaluation methods and commonsense aspects in a systematic way.

## 3 Methodology for searching relevant publications

To find publications suitable for answering our research questions RQ1–RQ4, we follow a structured, pre-defined procedure as proposed by Kitchenham and Charters (2007), Okoli (2015) and Page et al. (2021). To enhance the repeatability of our review, we create a review protocol containing additional information about the search as well as an overview of intermediate results. The protocol, as well as all additional artifacts, are available in our GitHub repository.

### 3.1 Applied search procedure

**FIGURE 1:**
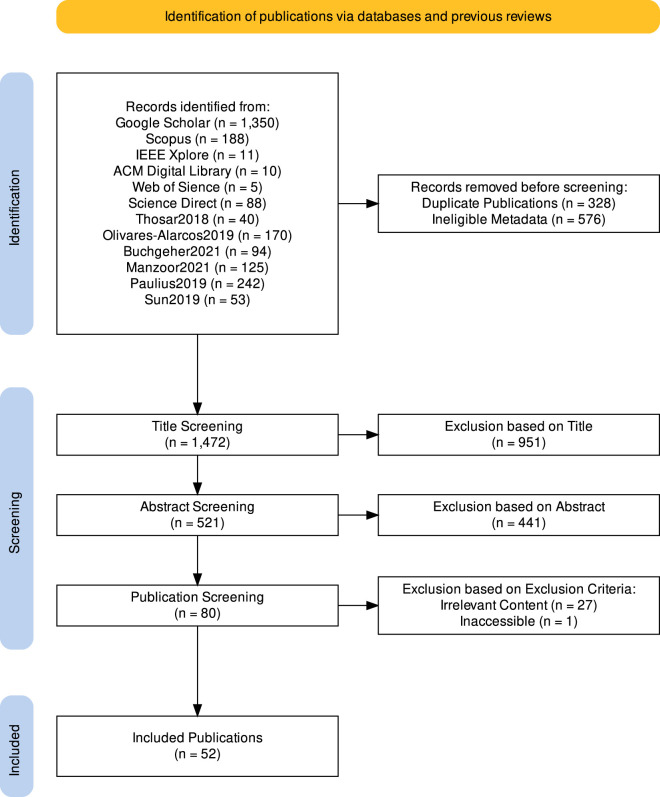
Visualizing our step-by-step search procedure and the number of publications found and analyzed in each step. This visualization was created with Haddaway et al. (2022).

To find publications suitable for answering our four research questions, we combine a keyword-based database search with a snowballing search on related surveys. The general procedure used, along with the quantity of publications identified and screened in each step, is visually summarized in Figure 1.

For the database search we defined the following four keywords before we started the search:

K1: “knowledge-enabled robot” OR “knowledge-based robot” OR “knowledge-driven robot”
K2: “knowledge processing” AND robot AND question AND NOT interaction AND NOT hardware
K3: “common sense knowledge” AND robot AND NOT interaction AND NOT hardware
K4: “common sense” AND (“robot cognition” OR “cognitive robot”)

We used each of these four keywords on the following six search engines/databases: Google Scholar, IEEE Xplore, Scopus, Web of Science, Science Direct and the ACM Digital Library. Through the combination of our keywords with these six sources, we found 1,652 publications.

Since we are not the first researchers to perform a literature review in the domain of knowledge representation and reasoning for cognitive robotics, we also decided to incorporate the results of previous published reviews. For this, we follow the guidelines by Wohlin (2014) for performing a snowballing search to gather publications that were either already covered by the reviews introduced in Section 2 or that cite these reviews. By collecting the in- and outgoing references of the reviews by Thosar et al. (2018), Olivares-Alarcos et al. (2019), Paulius and Sun (2019), Sun and Zhang (2019), Buchgeher et al. (2021) and Manzoor et al. (2021), we included 724 additional publications.

Combining the results of both search techniques yielded 328 duplicates, which we removed. We then analyzed the remaining publications regarding their metadata and removed 576 publications that did not fit the inclusion criteria described in Section 3.2. Next, we screened the 1,472 remaining publications in two steps, first looking only at their title, and then also covering their abstract. During these steps, we decided whether to include a publication using further steps based on the inclusion criteria specified in Section 3.2. This led us to exclude 951 publications based on their title and 441 based on their abstract, leaving us with 80 publications, which we read completely.

Of these 80 publications, one was not accessible in a full version, prompting us to exclude it as well. Of the remaining 79 publications, 27 were excluded based on the exclusion criteria described in Section 3.2, leaving us with 52 publications, which we analyzed to answer our research questions. A brief summary of these publications can be found in our review protocol.

### 3.2 Inclusion and exclusion criteria

To enhance the repeatability of our search, we define our inclusion and exclusion criteria before we start the search, as suggested by Kitchenham and Charters (2007). For the inclusion criteria, we differentiate between criteria regarding a publication’s metadata and its content. Regarding the metadata, we only include publications that were published in our investigated time frame of 11 years (i.e., between 2012 and 2022). For most of our data sources, these criteria were already applied during the search through explicit filters. Additionally, only papers that are written in English and thus understandable by the broad scientific community are included. Regarding the scientific quality, we focus only on publications that are peer-reviewed, excluding patents, books, presentations, technical reports and theses of any kind. Regarding the content, we analyze the title of the publication and its abstract in two separate steps to determine whether it contains a possible answer to any of our research questions. So, we include publications that discuss the application of CSK through a robot to a specific scenario or use case (RQ1), publications that discuss equipping cognitive robots with the possibility to answer certain CSK questions (RQ2), or that introduce or employ a (novel) source for collecting the necessary CSK (RQ3). In general, anything the authors employ as a source for gathering their CSK constitutes as an eligible resource for our analysis. This can cover texts, ontologies, websites, large language models or other kinds of data. Lastly, we do not define a specific inclusion criteria for assessing the evaluation methods and their used data (RQ4), since we expect all remaining publications to somehow evaluate their approach.

As we explain in Section 3.1, the exclusion criteria are applied after the metadata, title and abstract have already been analyzed. Here, we first exclude publications for which no complete version is available, thus making a thorough analysis impossible. Additionally, we exclude any publication we read completely but that turns out not to provide answers to any of our research questions, despite content in the title or abstract suggesting that it does.

## 4 Analyzing the usage of commonsense knowledge

**FIGURE 2:**
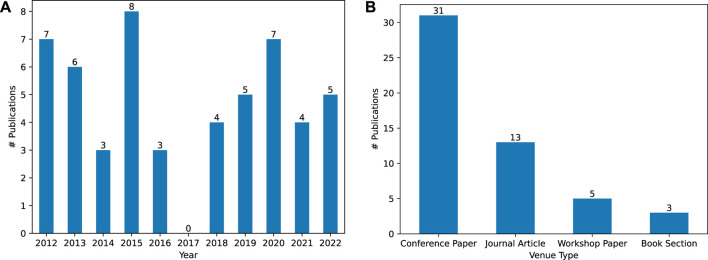
**(A)**: Visualizing the amount of found publications throughout the 10 year time span we restricted the search to. **(B)**: Visualizing the venue type where the found publications were published.

In this section, we analyze the content of the 52 publications found by the search procedure detailed in Section 3.1 to answer our four research questions introduced in Section 1. However, we first examine two aspects of their metadata. First, we examine the number of publications published for each year in our 11-year time span, visualized in Figure 2A. We do not find rising or falling trends in interest in the topic of CSK for cognitive robotics throughout these years, with a median of five publications per year. We also examine the venues where these publications were published. However, only three venues occur more than once: *Intelligent Service Robotics* ^2^ (2 occurrences), *IEEE International Conference on Robotics and Automation (ICRA)* ^3^ (6 occurrences) and *IEEE/RSJ International Conference on Intelligent Robots and Systems (IROS)* ^4^ (7 occurrences). For a more general examination, we summarize the venue type for all publications in Figure 2B. Here, we find that the majority of publications are conference papers (∼60%) , followed by journal articles (25%), workshop papers (∼10%) and lastly book sections (∼5%) .

### 4.1 Use cases and their application domain

Our first research question RQ1 pertains to the use cases for which the use of CSK has been considered in cognitive robotics research. When addressing this question, we differentiate between the concrete use case itself and the domain in which it is embedded. We look at both independently, since a given use case is not always embedded in a single domain. For example, the approach by Wang et al. (2019) focuses on the use case of finding and delivering a given object in the household domain, whereas Yang et al. (2019) focuses on the same use case but for the personal care domain.

**FIGURE 3:**
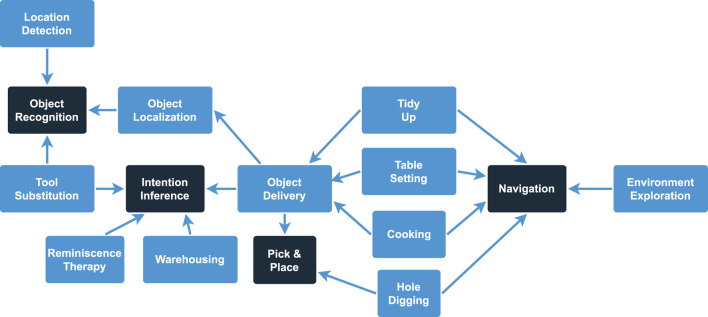
Visualizing the dependencies between the 15 different use cases. The four use cases in the darker rectangles are the low-level use cases that do not depend on any other use case.

For the distinction between possible domains and use case, we rely on the arguments and descriptions presented in each publication. The found domains are self-explanatory and mostly reported directly in each publication, so any publication that talks about the “household environment” counts towards the *Household* domain. For the use cases however, we collect their attributes and goals to distinguish and define the 15 different use cases seen below. Due to the difference in abstraction between these use cases, more complex use cases like, e.g., *Cooking* depend on other, more simplistic and low-level use cases like localizing or picking up objects. These dependencies are visualized in Figure 3.

• **Cooking** (Nyga and Beetz, 2012; Agostini et al., 2015): Generate and execute a cooking plan based on the current environment and a requested meal
•**Environment Exploration** (Pangercic et al., 2012; Kanjaruek et al., 2015; Jäger et al., 2018; Vassiliades et al., 2020; Zhang et al., 2021): Interacting with parts of the environment (objects, doors, cupboards, etc.) to gather (new) knowledge
•**Hole Digging** (Javed et al., 2016): Dig a hole in the garden
•**Intention Inference** (Liu et al., 2015; Liu and Zhang, 2016; De Silva et al., 2022): Identify the intention of a human with a certain object/command to react fittingly when the command cannot be executed (e.g., the robot should fetch the human some juice, which is not available. Why did the human want the juice and what is a fitting alternative?)
•**Location Detection** (Welke et al., 2013): Categorize the location based on the recognized objects (e.g., the robot detects milk and juice and concludes that the location is a fridge)
•**Navigation** (Shylaja et al., 2013; Li et al., 2022): Navigate to a specific location
•**Object Delivery** (Lam et al., 2012; Riazuelo et al., 2013; Mühlbacher and Steinbauer, 2014; Al-Moadhen et al., 2015; Zhang and Stone, 2015; Wang et al., 2019; Yang et al., 2019): Finding the requested object and delivering it to a specific location
•**Object Localization** (Varadarajan and Vincze, 2012b; Zhou et al., 2012; Kaiser et al., 2014; Riazuelo et al., 2015; Jebbara et al., 2018; Daruna et al., 2019; Zhang et al., 2019; Chernova et al., 2020): Finding a specific object in an (unknown) environment
•**Object Recognition** (Daoutis et al., 2012; Pratama et al., 2014; Kümpel et al., 2020; Chiatti et al., 2022): Recognize a specific object based on its properties
•**Pick and Place** (Al-Moadhen et al., 2013; Javia and Cimiano, 2016; Mitrevski et al., 2021): Pick an object up and place it at a different location
•**Reminiscence Therapy** (Wu et al., 2019): Asking questions about provided pictures to get the human to remember and socialize
•**Table Setting** (Salinas Pinacho et al., 2018; Haidu and Beetz, 2019): Set the table for a meal scenario (and maybe also clean up afterwards)
•**Tidy Up** (Aker et al., 2012; Skulkittiyut et al., 2013): Bring a specified part of the environment in order by removing unusual objects
•**Tool Substitution** (Zhu et al., 2015; Thosar et al., 2020; 2021; Dhanabalachandran et al., 2021; Xin et al., 2022): Recognizing a specific object as a suitable substitute for a missing tool
•**Warehousing** (Ayari et al., 2015; Pradeepani et al., 2022): Keep track of available objects and their quantity in an environment to inform a human once an object is unavailable

**FIGURE 4:**
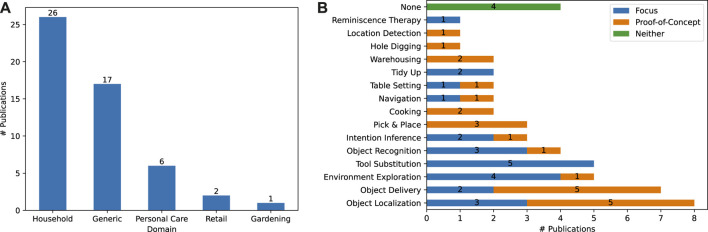
**(A)**: Visualizing the different domains in which the approaches operate. **(B)**: Visualizing the different use cases the approaches work on using CSK. We differentiate between publications that focus on their chosen use case or that use it as a proof-of-concept. A full explanation for each use case can be found in our review protocol.

During our analysis, we found five different domains where CSK is applied: the *Household* and *Retail* domains, the *Gardening* domain, the *Personal Care* domain, and the *Generic* domain in which the robot handles CSK in a way that can be applied to any other domain. The number of publications that handle each of these domains is visualized in Figure 4A. As can be seen, the *Household* domain is the focus of 50% of the covered publications, surpassing applications in the *Generic* domain (∼33%) . One commonality shared by the *Household*, *Personal Care* and *Retail* domains ( ∼65% of publications) is that robots operating in these domains potentially share their workspace with humans, which can lead to uncertainties in the environment that increase the need for robots to have and draw on CSK. Other domains where robots do not typically share their workspace with humans, such as industrial and manufacturing domains that tend to allow for better known and more deterministic environments, were not found during our analysis, despite the inclusion of approaches from these domains through the snowballing search on the reviews by Buchgeher et al. (2021) and Manzoor et al. (2021).

In addition to examining the application domain, we also investigate the specific use case with which each approach is concerned in Figure 4B. Here we distinguish between approaches that focus solely on a specific use case (e.g., Salinas Pinacho et al. (2018) focuses on the *Table Setting* use case) and approaches where a specific use case is used as an example or proof-of-concept to demonstrate the viability of the approach being proposed (e.g., Jebbara et al. (2018) use the *Object Localization* scenario to prove the applicability of their CSK extraction technique for the cognitive robotics domain). Roughly 46% of the analyzed publications (24 out of 52) focus on the use case they examine, whereas ∼46% use it only as an example application. The remaining four publications (Tenorth and Beetz, 2013; Beetz et al., 2018; Jakob et al., 2020; Beßler et al., 2022) have no specific use case, instead describing techniques intended to be generally applicable to the*Household* domain.

In general, use cases that focus on objects, and on their locations, affordances and relationships (*Object Localization, Object Delivery, Tool Substitution, Object Recognition, Pick and Place, Warehousing* and *Location Detection*) make up the majority of use cases, occurring in 30 out of 52 publications (∼58%) . Concrete household tasks like *Cooking, Tidying up* and *Table Setting*, which internally rely on the aforementioned object-focused use cases, are only covered in six publications (∼12%) . As we mentioned before, the majority of domains covered in our survey focus on environments that are shared by robots and humans. However, only four of the publications we analyzed cover direct interaction with humans through two use cases (*Intention Inference* and *Reminiscence Therapy*) (∼8%) .

### 4.2 (Un-)Answerable questions about commonsense knowledge

**FIGURE 5:**
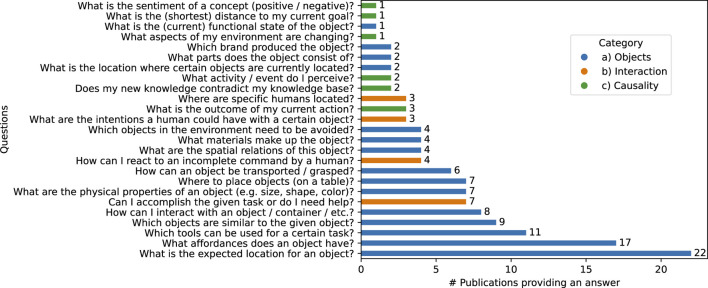
Visualizing the CSK questions and how many publications can provide an answer with their approach. Questions are split in three categories: **(A)** objects, their properties and relations, **(B)** intuitive psychology and human interaction or **(C)** intuitive physics and causality.

This section discusses the different commonsense questions for which the approaches discussed in the publications we analyzed can provide an answer (see RQ2). We gather these questions by analyzing the goals and capabilities of the approaches, keeping in mind the definition of CSK from Gupta and Kochenderfer (2004) provided in Section 1. This resulted in 25 different questions, which we separated into three categories: a) Objects, their properties and relations (e.g., *How can an object be transported/grasped?*), b) Intuitive psychology and human interaction (e.g., *What are the intentions a human could have with a certain object?*) and c) Intuitive physics and causality (e.g., *What is the outcome of my current action?*). We provide a visual summary of the 25 questions, their categories and the number of publications in which the discussed approach provides or proposes an answer in Figure 5A. The complete list of approaches that can answer each question is provided in the review protocol.

In general, the majority of questions, 15 out of 25 (60%), focus on objects, object properties and object relations. Looking at the number of approaches providing an answer, we discovered that 47 out of the 52 approaches (∼90%) can provide an answer to any question from this category. This heavy focus on objects is also represented in the most researched CSK questions, since eight out of the nine most answered questions in Figure 5B revolve around objects. Questions regarding intuitive psychology and human interaction are focused on in 14 out of 52 publications (∼27%) . However, these 12 publications concern themselves only with four different questions (16%). The remaining six questions all have to do with intuitive physics and causality (24%). However, only eight out of 52 publications try to achieve an answer to any questions in this category (∼15%) , a given publication often being the only approach that tries to answer the questions with which it is concerned (e.g., Shylaja et al. (2013) is the only approach answering the question *What aspects of my environment are changing?*).

Based on the aforementioned definition of CSK from Gupta and Kochenderfer (2004), we can provide some example questions that none of the 52 approaches analyzed are capable of answering. As we already observed, knowledge about intuitive physics and about intuitive psychology are not as well covered as knowledge about objects (see Figure 5A). Possible questions in these areas could be How can I (proactively) support the human in reaching their goals? or How do I handle objects based on their state of matter?. In addition to the more general object knowledge covered under the definition of Gupta and Kochenderfer (2004), more specific object properties only relevant for a specific use case/scenario are also investigated. This task-specific object knowledge is covered for the most frequently occurring use cases like Object Localization/Delivery or Tool Substitution. However, for more complex use cases like Cooking or Table Setting the necessary object knowledge to answer questions like How does this ingredient need to be processed to make it consumable? or What is a suitable table setup for a specific meal? are not covered.

It should be noted that it is possible that approaches outside of our analyzed set do cover some of these questions. However, our systematic approach lets us conclude that any such publication either does not apply its approach in the cognitive robotics domain or does not relate these questions to the keyword *commonsense knowledge*. We will talk about this divide in terminology in more detail in Section 5.

### 4.3 Sources for commonsense knowledge

To answer **RQ3**, we analyze the different knowledge sources employed by the analyzed publications. An overview of the 30 sources found and their properties can be examined in Table 1. To evaluate their relevance for the domain of cognitive robotics, we count the number of publications in which they occur. Additionally, we categorize them based on their type according to the criteria described by Hitzler et al. (Hitzler et al., 2010, Ch. 8.2, pp. 310-317). Lastly, we check whether the source is still available and can be downloaded and used.

**TABLE 1:** The 30 CSK sources employed by the 52 analyzed publications. Abbreviations in the **Type** column stand for *Structured (S)*, *Semi-Structured (SS)*, *Unstructured (U)* and *Human (H)* (Hitzler et al., 2010, Ch. 8.2, pp. 310-317).

Source	#	Type	Avail	Used by
ConceptNet Speer et al. (2017)	8	S	✓	Vassiliades et al. (2020); Aker et al. (2012) Skulkittiyut et al. (2013); Zhang et al. (2019) Jakob et al. (2020); Wu et al. (2019) Chernova et al. (2020); Kümpel et al. (2020)
Humans	7	H	✗	Ayari et al. (2015); De Silva et al. (2022) Haidu and Beetz. (2019); Lam et al. (2012) Liu and Zhang. (2016); Jakob et al. (2020) Nyga and Beetz. (2012)
Manually encoded	7	H	✗	Zhang and Stone. (2015); Yang et al. (2019) Beßler et al. (2022); Xin et al. (2022) Chiatti et al. (2022); Mitrevski et al. (2021) Dhanabalachandran et al. (2021)
OMICSGupta and Kochenderfer (2004)	6	SS	✗	Al-Moadhen et al. (2013); Al-Moadhen et al. (2015) Riazuelo et al. (2013); Riazuelo et al. (2015); Zhou et al. (2012) Nyga and Beetz. (2012)
(Open-)Cyc Lenat. (1995)	5	S	✓	Al-Moadhen et al. (2013); Al-Moadhen et al. (2015) Tenorth and Beetz. (2013); Daoutis et al. (2012) Mühlbacher and Steinbauer. (2014)
Perception/Sensors	5	U	✗	Ayari et al. (2015); Kanjaruek et al. (2015) Thosar et al. (2020); Thosar et al. (2021); Jäger et al. (2018)
WordNet Miller. (1995)	5	S	✓	Vassiliades et al. (2020); Kanjaruek et al. (2015) Skulkittiyut et al. (2013); Chernova et al. (2020) Nyga and Beetz. (2012)
Experience/Memories	4	H	✗	Shylaja et al. (2013); Pratama et al. (2014) Salinas Pinacho et al. (2018); Beetz et al. (2018)
Not mentioned	3	-	✗	Javed et al. (2016); Wang et al. (2019) Pangercic et al. (2012)
DBpedia Bizer et al. (2009)	2	S	✓	Jebbara et al. (2018); Vassiliades et al. (2020)
Google Books CorpusGoldberg and Orwant (2013)	2	U	✓	Kaiser et al. (2014); Welke et al. (2013)
Google Search Engine	2	U	✓	Skulkittiyut et al. (2013); Zhou et al. (2012)
WikiHow^a^	1	U	✓	Liu et al. (2015); Nyga and Beetz. (2012)
AfNet Varadarajan and Vincze. (2012a)	1	S	✓	Varadarajan and Vincze. (2012b)
AI2Thor Kolve et al. (2017)	1	SS	✓	Daruna et al. (2019)
BKN Lam et al. (2011)	1	S	✓	Lam et al. (2012)
BERT Devlin et al. (2019)	1	U	✓	Pradeepani et al. (2022)
Bing Image Search^b^	1	U	✓	Zhou et al. (2012)
Ehow Recipes^c^	1	U	✓	Kaiser et al. (2014)
FrameNet Baker et al. (1999)	1	S	✓	Nyga and Beetz. (2012)
KnowRob Tenorth and Beetz. (2013)	1	S	✓	Javia and Cimiano. (2016)
LabelMe Torralba et al. (2010)	1	SS	✓	Zhang et al. (2019)
Matterport3D Chang et al. (2017)	1	SS	✓	Li et al. (2022)
ShapeNet Chang et al. (2015)	1	S	✓	Chiatti et al. (2022)
TTU Dataset Zhu et al. (2015)	1	SS	✓	Zhu et al. (2015)
Unspecified Text	1	U	✗	Agostini et al. (2015)
Unspecified Images	1	U	✗	Xin et al. (2022)
Unspecified Videos	1	U	✗	Zhang et al. (2021)
VirtualHome Puig et al. (2018)	1	SS	✓	Vassiliades et al. (2020)
WikiData^d^	1	S	✓	Kümpel et al. (2020)

^a^ www.wikihow.com

^b^ www.bing.com/visualsearch

^c^ www.ehow.com

^d^ www.wikidata.org

Before analyzing the usage of these publications, we provide a quick overview over their capabilities:

• *ConceptNet* (Speer et al., 2017): ConceptNet is a semantic, multilingual network describing concepts through words and their commonsense relationships to each other. The necessary knowledge is collected through crowd-sourced resources, games with a purpose and resources created by experts.
• *OMICS* (Gupta and Kochenderfer, 2004): The *Open Mind Indoor Common Sense Project* is a collection of CSK for robots acting in the indoor domain (homes and offices). It collects knowledge in the form of statements, where each statement connects an object with an adjective describing either a property or the current object state.
• (*Open-)Cyc* (Lenat, 1995): Cyc provides users with a foundational/top-level ontology describing objects and actions through rules and assertions written by domain experts. OpenCyc and ResearchCyc describe two releases of this knowledge base that each contain a subset of all assertions.
• *WordNet* (Miller, 1995): WordNet provides a lexical database of the English language, where words are grouped into so-called *synsets* based on their semantics. Synsets are hierarchically structured using hyper- and hyponym relations as a foundation.
• *DBpedia* (Bizer et al., 2009): This project aims to extract structured information from Wikipedia by representing each entity through a unique identifier and its relationship to other entities.
• *Google Books Corpus* (Goldberg and Orwant, 2013): This corpus contains text from ∼3.5 million English books published between 1,520 and 2008. In addition, the authors provide a dataset containing all syntactic n-grams that can be extracted.
• *AfNet* (Varadarajan and Vincze, 2012a): The *Affordance Network* is a database containing structural and material affordances for common household objects. It is commonly employed for recognizing objects through their affordances.
• *AI2Thor* (Kolve et al., 2017): This dataset contains 3D indoor scenes that support many types of interaction for simulated robots. It consists of photo-realistic objects and scenes that can be procedurally generated.
• *BKN* (Lam et al., 2011): The *Basic-Level Knowledge Network* combines knowledge from children’s books, ConceptNet (Speer et al., 2017), and Google’s Web 1T 5-g corpus (Brants and Franz, 2006) in a knowledge base covering objects and activities. The focus of this knowledge base lies in providing answers to *Where*, *What*, and *How* questions.
• *BERT* (Devlin et al., 2019): *Bidirectional Encoder Representations from Transformers* describes a family of large language models that are pre-trained on a corpus of unlabeled text and can be fine-tuned to fit the purpose of the task.
• *FrameNet* (Baker et al., 1998): Lexical database of concepts embedded in their semantic frame to better understand the concept’s meaning.
• *KnowRob* (Tenorth and Beetz, 2013): This knowledge processing system is employed for automated robots and formulates decisions a robot can make as inference tasks that can be answered by virtual knowledge bases (KBs). These KBs combine word meaning from WordNet (Miller, 1995) with OpenCyc (Lenat, 1995), gather object information from online shops and contain observed human behavior.
• *LabelMe* (Torralba et al., 2010): This database contains annotated images focusing on objects, scenes and their spatial connection. The annotations were provided by volunteers using an online annotation tool. Through this tool, the database accumulated over 400,000 annotations.
• *Matterport3D* (Chang et al., 2017): Matterport3D is a large-scale dataset containing panoramic views made up of different images and taken in different buildings. Additional annotations describe information about camera poses and semantic segmentation.
• *ShapeNet* (Chang et al., 2015): This is a richly annotated, large-scale dataset containing 3D models for different household objects collected from public repositories and other existing datasets. The objects are categorized on the basis of their corresponding synset in WordNet (Miller, 1995).
• *TTU Dataset* (Zhu et al., 2015): The *Tool and Tool-Use* dataset is used for evaluating the recognition of tools and task-oriented objects by providing a collection of static 3D objects. These objects are combined with a set of human demonstrations regarding their usage.
• *VirtualHome* (Puig et al., 2018): The VirtualHome simulator uses a crowd-sourced knowledge base of household tasks, represented through a name and a list of instructions. These instructions are translated into program code that is executed in a simulated 3D environment by virtual agents.

In general, we do not find one source that is predominantly used. Even ConceptNet (Speer et al., 2017), which has the most usage in our data, is only employed by roughly 15% of publications. Similarly, 17 out of the 30 sources (∼57%) we found are only employed by a single publication, which demonstrates that most publications use specialized sources for the specific scenarios they work in rather than relying on a single, more general source. However, even when we focus on a specific use case we do not find a single source on which all approaches rely. This is underlined by the summary of CSK sources per use case provided in Table 2. As described in that table, there is no source that is used more than two times for a specific use case, with most sources occurring only once per use case. This demonstrates that none of the 28 sources provides data specific for a single use case but all of them focus on aspects relevant for different use cases.

**TABLE 2:** Summary of the 15 use cases we found and the sources that are employed to gather commonsense knowledge for the specific use case. The *Hole Digging* use case is omitted since it is only discussed in a single publication that does not mention a source (Javed et al., 2016).

Use case	Employed sources
Object Localization	ConceptNet (2x), OMICS (2x), AI2Thor, AfNet, Bing Image SearchDBpedia, Ehow Recipes, Google Books Corpus, Google Search EngineLabelMe, WordNet
Object Delivery	(Open-)Cyc (2x), OMICS (2x), BKN, Humans
Environment Exploration	Perception/Sensors (2x), WordNet (2x), ConceptNet, DBpediaUnspecified Videos, VirtualHome
Tool Substitution	Perception/Sensors (2x), TTU Dataset, Unspecified Images
Intention Inference	Humans (2x), WikiHow
Object Recognition	(Open-)Cyc, ConceptNet, Experience/Memories, ShapeNet, WikiData
Navigation	Experience/Memories, Matterport3D
Pick and Place	(Open-)Cyc, KnowRob, OMICS
Table Setting	Experience/Memories, Humans
Tidy Up	ConceptNet (2x), Google Search Engine, WordNet
Warehousing	BERT, Humans, Perception/Sensors
Cooking	FrameNet, Humans, OMICS, Unspecified Text, WikiHow, WordNet
Location Detection	Google Books Corpus
Reminiscence Therapy	ConceptNet

**FIGURE 6:**
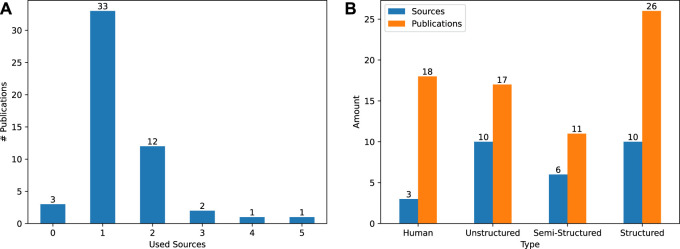
**(A)**: Visualizing the number of different sources used by each publication. Across all 52 publications, 30 different CSK sources were employed. **(B)**: Visualizing the types of sources (Hitzler et al., 2010, Ch. 8.2, pp. 310-317), their amount and the number of their occurrences throughout the 52 different publications.

In addition to looking at which sources are employed, we also count the number of sources each publication relies on. Here we found that the majority of publications (33 out of 52, ∼63% ) relies on a single source for extracting its CSK. Only 16 publications (∼31%) combine two or more sources, either to cover a broader scope of CSK (e.g., Kümpel et al., 2020) or to increase the quality of the data extracted (e.g., Vassiliades et al., 2020). The described results are visualized in Figure 6A.

Regarding the type of source, we count the number of sources per type and the number of publications employing this type in Figure 6B. Only three sources (∼11%) depend on knowledge provided by human domain experts. However, these sources are applied in 35% of publications. The same amount of structured as well as unstructured sources (10 out of 30, ∼ 33%) are used according to our data. However, the ten structured sources are employed the most by the publications (∼38%). In general, the high reliance on structured sources is a positive development, since sources of this type are formalized to enhance machine readability.

Despite four approaches that extract CSK from unstructured text (Welke et al., 2013; Kaiser et al., 2014; Agostini et al., 2015; Liu et al., 2015) using NLP techniques, only the approach by Pradeepani et al. (2022) employs a large language model (Devlin et al., 2019) as its data source. Since research on large language models is a rather new domain, approaches that connect them with robots are still scarce (e.g., Ahn et al., 2022) and not yet focused on CSK. This supports the recommendation formulated by Wray et al. (2021) that there needs to be further research to increase the suitability of these models for the cognitive robotics domain.

Lastly, we briefly want to touch on additional sources that are not employed for the extraction of CSK for cognitive robotics. In a recent survey on CSK sources by Ilievski et al. (2021a), 22 different resources were collected and evaluated. However, only four sources found by our analysis overlap with these resources from their study (ConceptNet, WordNet, FrameNet and Wikidata), making up only 15 of the 75 CSK source usages (20%). So the remaining 18 sources have yet to be applied to the cognitive robotics domain.

### 4.4 Evaluation methods and benchmarking

To answer our last research question and investigate which methods and which datasets are used by the 52 collected approaches during their evaluation, we adapt the evaluation method taxonomy presented by Konersmann et al. (2022) for the software architecture domain. In general, not all methods are applicable to the cognitive robotics domain. In our data, we found *Motivating Examples (Technical) Experiments and Case Studies*. Additionally, we add the method *Model Evaluation* for approaches that evaluate an ML model without connecting it to a simulated or real-world robot. For the two most common methods, *Experiments* and *Case Studies*, we additionally differentiate whether they are performed in the real-world using a real robot or if the robot is simulated and operates in a simulated environment.

**FIGURE 7:**
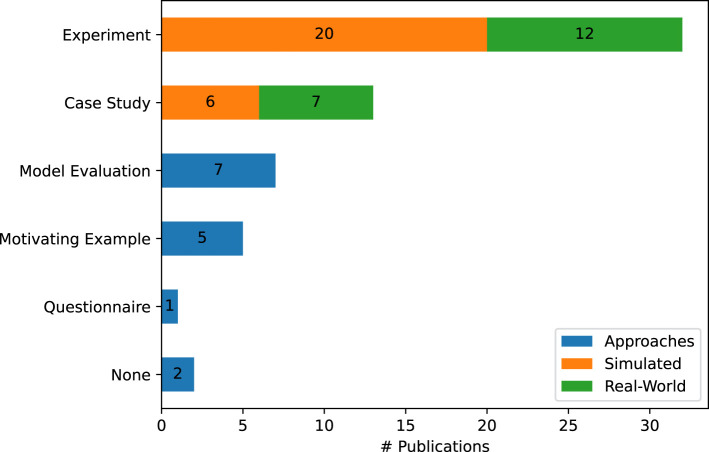
The different evaluation methods in our 52 analyzed publications. For the *Case Study* and *Experiment* we differentiate between a simulated or a real-world environment and robot.

The resulting occurrences can be examined in Figure 7. In general, the majority of approaches (∼62%) are evaluated using a quantitative experiment, with most of these approaches being done in a simulated environment (∼63%) . Generally, simulation environments are used in 50% of publications whereas evaluation on a real robot is performed in only ∼37% of publications.

In addition to the evaluation method, we also gather information regarding the data that was used for the evaluation, as well as its availability. Here, we find that 32 out of the 52 publications (∼62%) did not publish the data used for the evaluation, and four publications (∼8%) did not use any data for their evaluation. In the 16 remaining publications, only two datasets, are used more than once. AI2Thor (Kolve et al., 2017) is used in Daruna et al. (2019); Li et al. (2022) and Al-Moadhen et al. (Al-Moadhen et al., 2013; Al-Moadhen et al., 2015) both use the same basic example household, described in either publication. Except for two employed datasets, all of the employed datasets are still available either online or by being directly provided in the publication.

These findings notwithstanding, we recognize that in the cognitive robotics domain, a correct execution of the desired task without the occurrence of unwanted side effects can be regarded as a proper and successful evaluation of an approach Vernon et al. (2022). Since the execution environment and the robot programs are often very specific to the lab where they are programmed, there are additional challenges that come with making them publicly accessible (Gu et al., 2023). However, there are certain aspects of CSK for the cognitive robotics domain where benchmarking makes sense. For example, the main question from Section 4.2 *What is the expected location for an object?* is often evaluated by comparing the (automatically) generated locations to a gold standard. However, this gold standard is often not taken from a publicly available dataset, but is instead created by the authors. In general, we observe a lack of benchmarks for domain-specific CSK questions like this.

## 5 Discussion

Our analysis of the selected publications has revealed interesting limitations and gaps in the way commonsense knowledge is currently used in cognitive robotics research. First of all, while there are many potential use case and applications where commonsense knowledge might support generalization, our analysis has revealed a strong focus on use cases related to acquiring knowledge about objects in order to support things such as object localization and delivery, tool substitutions or pick and place.

This focus on object knowledge is understandable as knowledge about objects to a large extent comprises of static knowledge related to the properties and characteristics of objects, which lend itself to being modeled using the state-of-the-art graph-based knowledge representation language that can straightforwardly model (relational) knowledge about objects using edges or triples. Modeling knowledge about events, their logical and causal structures requires more complex representational paradigms. Further, there are less commonsense knowledge sources containing event knowledge compared to data sources containing (relational) knowledge about concepts and/or objects.

Regarding the sources of commonsense knowledge used, we observe quite a diversity and spread with many different sources being used. This shows that the field seems to be in an experimental state, testing different resources, without clear best practices having emerged. There seem to be no integrative resources that contain all sorts of relevant knowledge so that in the future we can expect that no source will fit all purposes and that robotic systems will have to rely on a combination of sources for different tasks and purposes.

In terms of evaluation and domains, we observe a clear focus on service robotics scenarios and household applications in contrast to the application of robotic systems in industry or production. The explanation for this seems quite natural: industrial settings have less variance and require that the same task is executed over and over with accuracy and precision. In such scenarios there is much less uncertainty than in scenarios where a robot might be confronted with new and unknown tasks, objects, situations, etc. As robots can not be pre-programmed to handle all these situations, flexible reasoning based on commonsense knowledge seems key to master the variance and uncertainty characteristics of such more open environments.

Finally, the lack of focus on physical reasoning and psychological reasoning in terms of applications is understandable, as these types of tasks require commonsense knowledge in the sense of having the ability to simulate physical environments or simulate others to infer their intentions, goals, etc. The first one requires accurate physics engines that would allow a robot to make accurate (forward) predictions. The latter one would require modules to make inferences about other agents, a so called computational Theory of Mind (ToM), the realization of which is a complex and long-term challenge Lake et al. (2017).

As we have seen above, knowledge about objects plays a central role. This is clearly related to the notion of affordances that is studied in cognitive robotics literature, as surveyed by Min et al. (2016). In these approaches, the affordances for the environment and its objects are learned, mostly by using machine learning-based methods on images or videos. What is striking here is that there seems to be a terminological gap in the way the semantic technology or knowledge representation community conceptualize object knowledge and how it is represented. While the semantic tech and KR communities often focus on (static) object knowledge, the cog. rob community focuses on perceptually grounded and action-related knowledge, thus using the concept of ‘affordances’ that indicate an action potential.

To further examine this divide in terminology, we examine the classification performed by Zech et al. (2019) in their review, which we introduced in Section 2. Despite focusing on actions and their possible representation in the cognitive robotics domain, their classification does not connect to keywords associated with the knowledge representation and reasoning community such as *ontology*. Similarly, there are concepts that are handled in Zech et al.‘s classification, but with a different focus/level of detail than in the publications we analyzed. As an example, we look at the concept of *affordance*. In the classification schema proposed by Zech et al., an affordance is given as an example of an *exteroceptive stimuli*, which is a stimuli generated in the external environment to provide interaction possibilities (Zech et al., 2019). In our analyzed publications, an affordance is defined as either 1) “a relation of an action/activity/intention and a specific object used to predict the next action/activity” (Liu et al., 2015, p. 1962) or 2) “the relational context holding between several objects that play different roles” (Beßler et al., 2022, p. 8). If we compare these three characterizations of *affordance*, we see that the one by Zech et al. focuses on the immediate application of this concept for robotic action execution, whereas definitions 1) and 2) focus more on the knowledge that an affordance can provide the robot to support, e.g., the planning of future steps or an understanding of the semantic similarities between different objects and actions. Another example is the concept of *intuitive physics*, which we introduced as one part of the definition for CSK in Section 1. This concept has no direct representation in the classification schema by Zech et al., despite its relevance for a successful action execution. The closest concept is *effect associativity*, which analyses whether a representation covers predicting the effect of an action based on its description.

The generalization of task execution knowledge is an important problem in current cognitive robotics research. To allow robots to be employed in domains shared with humans, robots need to be able to handle underspecified commands for manipulating unknown objects in an unknown way in a dynamic environment. Publications like the ones covered by our review and by Zech et al. (2019) are all trying to solve aspects of this generalization problem, despite coming from different research communities and often using different tools and approaches. This difference is underlined by the fact that there is no overlap between the 52 publications included in our study and the 152 publications included in Zech et al. (2019). In the future, more collaboration is needed to bridge this divide between the two communities, if we are to successfully tackle the task generalization problem.

## 6 Threats to validity

In general, we integrate different countermeasures into our process by following the general process for systematic literature reviews by Kitchenham and Charters (2007). However, there still remain some biases that we can not completely prevent. To address these threats, we examine selection, measurement and exclusion bias as well as repeatability separately.

**Selection Bias:** We have selection bias since the insights we gained through the paper analysis depend on the subset of papers we chose. Despite including all 52 publications we deemed suitable for answering our research questions, this inclusion is still based on pre-defined inclusion and exclusion criteria. These were not chosen randomly but derived from our research questions and the search procedure recommendations from Kitchenham and Charters (2007); Okoli (2015); Page et al. (2021).

**Measurement Bias:** Another problem is measurement bias, since the screening of the search publications was carried out by one of the authors. As a countermeasure, we pre-defined the set of inclusion and exclusion criteria before beginning the search. However, the filtering is still prone to human errors.

**Exclusion Bias:** Another possible problem stems from the exclusion of potentially interesting publications. By starting our systematic search with recent review papers in cognitive robotics, we have introduced this bias as research in AI, cognitive science, language processing, and cognitive robotics is still not sufficiently connected.

To counter this threat, we pre-defined the criteria we use for including and excluding publications. They are chosen to be as fitting for our research questions as possible and to not hinder the quality of our results. Additionally, no adjustments were made during the screening process. This prevents the exclusion of publications that were initially chosen but then excluded due to a failure to fit results during the analysis.

**Repeatability:** As the name suggests, threats to this validity describe problems encountered when other researchers try to emulate and repeat this evaluation. To allow for the repetition of the case study, we document all decisions, such as the inclusion and exclusion criteria, the keywords and search engines, in our review protocol. Additionally, all artefacts we created during our review are available in the aforementioned GitHub repository. However, the repeatability of our study is also limited due to the fact that only one person was responsible for screening the search results.

## 7 Conclusion and future work

In this article, we have investigated the coverage of CSK in the cognitive robotics domain by evaluating the use cases and domains for which CSK is used, the aspects of CSK that are addressed, the sources employed for gathering the necessary CSK and the method of evaluation. For this purpose, we performed a systematic literature review using a keyword search on six search engines combined with a snowballing search on six related reviews. The resulting 2,048 publications were screened and filtered, which left us with 52 publications deemed suitable for answering our research questions.

By reviewing these 52 publications, we found that most use cases occur in the household domain and focus on objects and their relations to the environment, especially their location. This was corroborated by looking at what sorts of questions CSK are called upon to answer. We found that the most common CSK questions seek to connect an object to a specific location in its environment. Other important questions focus on object similarity, object affordances and tool substitution. Generally, questions focusing on objects are much more dominant than questions about interacting with humans or about physics or causality of actions. Regarding the employed sources, we found that specific sources like ConceptNet (Speer et al., 2017) (Open-)Cyc (Lenat, 1995) or OMICS (Gupta and Kochenderfer, 2004) are used in multiple publications but there is not one single source that covers all relevant aspects of CSK. Similarly, there are often multiple sources used to answer the same CSK questions. Regarding the evaluation performed in these publications, we also found that there are few resources used as data and most of the publications do not publish their evaluation data. This lack of available benchmarks and datasets is surprising since most the publications are evaluated using either a case study or an experiment, which both are mostly performed in simulation, thus leading to a high amount of data necessary for a successful execution. However, only a small amount of publication publish this data.

This review’s limitations stem from the threats to validity described in Section 6. In general, we counteract most threats by following the guidelines in Kitchenham and Charters (2007); Okoli (2015); Page et al. (2021) and documenting our decisions and intermediate steps in the reviewprotocol. The main limitation is the data analysis, which was manually performed by a single person.

Lastly, in our discussion of the review by Zech et al. (2019) we emphasized a terminological gap that exist between communities, the knowledge representation community on the one hand and cognitive robotics community on the other hand. These terminological differences need to be bridged towards developing an interdisciplinary research community that synergistically brings together the different aspects of commonsense and makes them actionable in robot control systems.

In the future, focus should lie on the evaluation and benchmarking of commonsense aspects for the cognitive robotics domain, as we explored in Section 4.4. For this, we want to investigate the applicability of commonsense reasoning benchmarks (e.g., CommonsenseQA Talmor et al. (2019)) for the cognitive robotics domain by evaluating their coverage of the relevant aspects we presented in Section 4.2. Additionally, as we explained in Section 4.3, there are different CSK datasets and resources from the survey by Ilievski et al. (2021a), who have yet to be applied to the cognitive robotics domain. This also includes new resources that have been published since the aforementioned study, like the *CommonSense Knowledge Graph (CSKG)* (Ilievski et al., 2021b) or *Ascent++* (Nguyen et al., 2022). Finally, considerable focus should be put on creating the aforementioned interdisciplinary research community.

## Data Availability

The datasets presented in this study can be found in online repositories. The names of the repository/repositories and accession number(s) can be found below: https://github.com/ag-sc/Robot-Commonsense-Review.

## Footnotes

1
https://github.com/ag-sc/Robot-Commonsense-Review

2
www.springer.com/journal/11370

3
www.ieee-ras.org/conferences-workshops/fully-sponsored/icra

4
www.ieee-iros.org
